# Calpain-2 Regulates Kinesin and Dynein Dysfunction in Neurotoxin-Induced Motoneuron Injury

**DOI:** 10.3390/brainsci16010092

**Published:** 2026-01-16

**Authors:** Vandana Zaman, Camille Green, Kayce Sitgreaves, Amy Gathings, Kelsey P. Drasites, Noah Coleman, Jessica Huell, Townsend McDonald, Narendra L. Banik, Azizul Haque

**Affiliations:** 1 Ralph H. Johnson Veterans Administration Medical Center, 109 Bee Street, Charleston, SC 29401, USA; greecami@musc.edu (C.G.); kab319@musc.edu (K.S.); mcdonato@musc.edu (T.M.); baniknl@musc.edu (N.L.B.); 2 Department of Neurosurgery, Medical University of South Carolina, 96 Jonathan Lucas Street, Charleston, SC 29485, USAkelseydrasites@mail.rossmed.edu (K.P.D.); colemano@musc.edu (N.C.); huellj@musc.edu (J.H.); 3 Department of Pharmacology and Immunology, Medical University of South Carolina, 173 Ashley Avenue, Charleston, SC 29425, USA; 4 Hollings Cancer Center, Medical University of South Carolina, 86 Jonathan Lucas Street, Charleston, SC 29425, USA

**Keywords:** dynein, kinesin, Parkinson’s disease, calpain, reactive oxygen species, spinal cord, neurodegeneration

## Abstract

**Background/Objectives:** Neurodegenerative diseases are driven by multiple interconnected pathological mechanisms involving both intrinsic and extrinsic molecular and cellular processes. Efficient bidirectional intracellular transport is essential for neuronal survival and function, enabling the movement of organelles, proteins, and vesicles between the neuronal soma and distal compartments. This process is primarily mediated by kinesin-dependent anterograde transport and dynein-dependent retrograde transport. Disruption of either motor protein compromises endosome–lysosome recycling, leading to cellular dysfunction and neurodegeneration. However, the mechanisms underlying motor protein impairment in Parkinson’s disease (PD) remain incompletely understood. **Methods:** We investigated the involvement of kinesin and dynein in intracellular transport dysfunction using both in vitro and in vivo models of PD. Cultured neuronal cells were exposed to MPP+ (1-methyl-4-phenylpyridinium) to model PD-associated neurotoxicity, and motor protein function, vesicular trafficking, and endosomal recycling were assessed. In parallel, an MPTP (1-methyl-4-phenyl-1,2,3,6-tetrahydropyridine)-induced mouse model of PD was used to evaluate dynein-positive fiber density in the spinal cord. The role of calpain-2 was examined by co-treatment with the selective calpain-2 inhibitor zLLYCH2F in both experimental systems. **Results:** MPP+ exposure disrupted kinesin- and dynein-mediated transport in neuronal cytoplasm, resulting in impaired vesicular trafficking and defective endosome–lysosome recycling. These alterations led to abnormal accumulation of vesicles in both perinuclear regions and at the cell periphery. Pharmacological inhibition of calpain-2 with zLLYCH2F restored motor protein function and normalized vesicle distribution in MPP+-treated cells. Consistent with in vitro findings, MPTP-treated mice exhibited a significant reduction in dynein-positive fiber density within the spinal cord, which was prevented by co-treatment with zLLYCH2F. **Conclusions:** Our findings demonstrate that calpain-2 activation contributes to kinesin and dynein dysfunction following MPP+/MPTP exposure, leading to impaired intracellular transport and vesicle recycling in PD models. Inhibition of calpain-2 preserves motor protein function, maintains cytoskeletal integrity, and supports normal intracellular trafficking. These results identify calpain-2 as a critical regulator of motor protein stability and suggest that targeting calpain-2 may represent a promising therapeutic strategy for mitigating intracellular transport defects in Parkinson’s disease.

## 1. Introduction

Parkinson’s disease (PD) is a progressive neurological disorder caused by the ongoing loss of dopamine-producing neurons in the substantia nigra region of the brain [1,2,3]. This loss reduces dopamine signaling in the striatum and leads to the motor symptoms that define the disease [4,5]. However, PD is not limited to the nigrostriatal pathway; degeneration also occurs in other regions of the nervous system, including the spinal cord, which contributes to worsening movement problems and cognitive decline as the disease progresses [6,7,8]. Current treatments aimed at reducing mitochondrial damage or oxidative stress have shown limited effectiveness, and long-term dopamine replacement therapy may worsen symptoms in later stages of PD [1,8]. A growing body of evidence indicates that chronic inflammation is a significant contributor to the progression of PD [6,7]. This process involves the activation of glial cells, such as microglia and astrocytes, which release inflammatory factors capable of damaging neurons in both the brain and spinal cord [6,7]. A central mediator in this inflammatory cascade is calpain, a calcium-dependent protease whose activity becomes elevated under inflammatory conditions. Notably, heightened calpain activity has been observed in postmortem PD brain and spinal cord tissues, as well as in various experimental models of the disease [9,10,11,12]. Consequently, sustained inflammation driven by calpain may constitute a key mechanism responsible for the degeneration of dopaminergic and motor neurons in PD. Notably, calpain activation can directly compromise axonal transport by proteolytically targeting cytoskeletal and motor proteins, including kinesin and dynein, thereby disrupting bidirectional cargo trafficking essential for neuronal survival. This convergence of inflammation and transport failure positions calpain-dependent motor protein dysfunction as a unifying mechanism contributing to neurotoxin-induced motoneuron injury in PD.

Axonal transport is a fundamental process required for neuronal maintenance, synaptic communication, and survival [13,14]. Motor neurons are particularly vulnerable to impairments in this system due to their long axonal projections and reliance on rapid, continuous trafficking of organelles, cargoes, and signaling vesicles between the soma and distal terminals [13,15,16]. This transport depends primarily on the coordinated activity of kinesin and dynein, which mediate anterograde and retrograde trafficking, respectively. Disruption of these motor proteins has been implicated in several neurodegenerative disorders, including amyotrophic lateral sclerosis (ALS), PD, and peripheral neuropathies [17,18,19,20]. Among the models used to investigate such pathologies, the neurotoxins MPTP (1-methyl-4-phenyl-1,2,3,6-tetrahydropyridine) and its active metabolite MPP^+^ remain widely employed for their ability to induce dopaminergic and non-dopaminergic neuronal injury through mitochondrial dysfunction, ATP depletion, reactive oxygen species (ROS) accumulation, and cytoskeletal disorganization [21,22,23,24].

Although MPP^+^/MPTP-induced defects in axonal transport have been studied primarily in dopaminergic neurons [21,25,26], less is known about how these toxins affect spinal motoneurons, which are integral for motor function and may undergo transport-related degeneration in multiple neurological conditions. Moreover, the molecular mediators linking MPP^+^-driven oxidative stress to alterations in transport machinery remain only partially defined. One promising candidate is calpain-2, a Ca^2+^-dependent cysteine protease known to cleave cytoskeletal substrates, disrupt microtubule stability, and potentiate neurodegenerative signaling cascades [27,28,29]. Calpain-2 activation has been implicated in axonal degeneration, impaired neurite outgrowth, synaptic dysfunction, and vesicular transport abnormalities across various disease models [29,30]. However, the precise contribution of calpain-2 to MPP^+^/MPTP-induced motoneuron pathology, particularly regarding motor protein redistribution and transport deficits, has not been fully elucidated.

The rationale for targeting calpain-2 stems from its dual involvement in both acute cellular stress responses and progressive neurodegenerative mechanisms. Excessive calpain-2 activation has been shown to degrade key structural proteins, alter growth cone dynamics, and contribute to axonal retraction [31,32,33], making it a compelling therapeutic target to rescue neuronal architecture and function. Inhibition of calpain-2 may therefore preserve cytoskeletal integrity, maintain normal motor protein localization, and promote recovery of axonal transport under toxic conditions. Given that impaired vesicle trafficking is an early and potentially reversible event in neurodegeneration [34,35], identifying strategies that prevent transport failure holds substantial clinical significance.

The present study investigates these mechanisms using both in vitro and in vivo models. In VSC 4.1 motoneuron cells, we evaluated how selective calpain-2 inhibition modulates neurite morphology, ROS production, and the subcellular distribution of kinesin and dynein following IFN-γ or MPP^+^ exposure. We further assessed whether these protective effects translate to spinal cord tissue by examining motor protein fiber density in mice treated with MPTP alone or in combination with a calpain-2 inhibitor. This dual approach enables a comprehensive evaluation of calpain-2’s role in regulating intracellular transport pathways. By integrating quantitative imaging, biochemical analysis, and neurotoxic modeling, this work identifies calpain-2 as a key mediator of motor protein dysfunction and highlights its inhibition as a promising strategy for preserving neuronal transport systems under neurotoxic stress.

Overall, these findings address an important knowledge gap concerning the molecular underpinnings of transport failure in motoneurons and provide mechanistic insight into how calpain-2 inhibition may counteract early degenerative processes. Understanding these relationships is critical for developing therapeutic interventions aimed at stabilizing axonal transport, an emerging and potentially disease-modifying target in neurodegeneration.

## 2. Materials and Methods

### 2.1. In Vitro Studies

#### 2.1.1. Cell Culture

The VSC4.1 cell line (a hybrid cell line resembling spinal motor neurons) [36] was used to study how blocking calpain-2 affects cell survival and the function of important motor proteins. These cells were grown in Dulbecco’s Modified Eagle’s Medium (DMEM)/Hams F-12 50/50 mix with L-glutamine (cat# 10-090-CV Corning, Manassas, VA, USA), 2% Bovine Calf Serum (cat# SH30087.03, Hyclone, Logan, UT, USA) and 2% SATO’s 50× Solution [37]. Falcon 4-well culture slides (cat# 354104 Corning, Manassas, VA, USA), were coated with Poly-L-Ornithine (cat# A004 C, Sigma, St. Louis, MO, USA) for one hour at room temperature and rinsed with sterile PBS before adding 500 uL of VSC4.1 cells suspended in complete media into each well [37,38]. All cultures were kept in a standard cell-culture incubator at 37 °C with 5% CO_2_ and high humidity, which provides a stable environment for cell growth.

#### 2.1.2. MTS Cell Viability Assay

To determine the optimum concentration of MPP^+^ (Sigma, St. Louis, MO, USA, cat# D048) and calpain-2 inhibitor (zLLYCH_2_F, cat# A 6060, Sigma-Aldrich, St. Louis, MO, USA) in in vitro experiments, we first measured cell viability using the MTS assay (CellTiter 96 Aqueous One Solution, cat# G3581 Promega, Madison, WI, USA) [39]. This test measures how many cells are alive based on their ability to convert the MTS reagent into a colored product.

VSC4.1 cells were prepared at a density of 5 × 10^5^ cells/mL, and 100 µL of this suspension was added to each well of a 96-well plate. Cells were treated with 50 or 100 µM of the MPP+ toxin, while in another set of experiments, the cells were treated with various concentrations of the calpain-2 inhibitor (1, 5, 10, 20, and 30 µg/mL). Cells were then incubated with these treatments for 24 h. After this period, 20 µL of the MTS reagent was added to each well and incubated at 37 °C. The reagent produces a color change that reflects metabolic activity, and the amount of color is measured as absorbance at 490 nm using a plate reader (BioTek EL800, Agilent, Santa Clara, CA, USA). The absorbance was measured twice, after 30 min and again after an additional 30 min, to ensure accurate detection. The entire assay was repeated at least three times, and data are shown as mean cell viability ± standard deviation.

#### 2.1.3. Reactive Oxygen Species (ROS) Assay

Because oxidative stress contributes to neuronal injury, we next tested whether the calpain-2 inhibitor could reduce ROS production [40]. VSC4.1 cells were grown in T75 flasks until they reached ~80% confluency, harvested, and counted. Cells were seeded at 5 × 10^4^ cells per well in black-walled, clear-bottom plates to allow fluorescence detection.

To mimic inflammation, cells were treated with interferon-gamma (IFN-γ, 40 ng/mL). Four treatment conditions were tested in triplicate: (1) Control (vehicle: 0.01% DMSO), (2) IFN-γ alone, (3) calpain-2 inhibitor alone (10 and 20 µg/mL), and (4) IFN-γ + calpain-2 inhibitor 10 and 20 µg/mL). The total volume in each well was 200 µL. ROS levels were measured after 24 hrs of treatments using the Abcam ROS Assay Kit (cat# ab186029 Abcam, Cambridge, MA, USA), which uses a fluorescent probe that lights up when oxidized by reactive oxygen species. Measurements were taken using Molecular Devices SpectraMax iD3 Plate Reader (Molecular Devices, LLC, San Jose, CA, USA) following the manufacturer’s instructions. Statistical comparisons were made using a two-tailed *t*-test.

#### 2.1.4. Immunocytochemistry

To examine how calpain-2 inhibition affects motor proteins that are essential for transporting materials inside neurons, we performed immunostaining for dynein and kinesin [38]. VSC4.1 cells were plated onto poly-L-ornithine-coated four-chamber slides at a density of 150,000 cells/mL (500 µL per well). Cells were treated for 20–24 h with one of the following: (1) Saline (control), (2) MPP^+^ (100 µM), a neurotoxin that damages dopaminergic and motor neurons, and (3) MPP^+^ (100 µM) + calpain-2 inhibitor (zLLYCH_2_F, 20 µg/mL).

After treatment, cells were fixed in ice-cold methanol to preserve their structure. Slides were then blocked in 8% normal horse serum for one hour at room temperature and stained with primary antibodies against MAP2 rabbit polyclonal (1:500, AB5622 Sigma-Aldrich), Dynein mouse monoclonal (1:500, cat# MA1-070 ThermoFisher Scientific, Carlsbad, CA, USA), or Kinesin mouse monoclonal (1:500, sc-271471 Santa Cruz Biotechnology Inc., Dallas, TX, USA). After overnight incubation at 4 °C, slides were washed and incubated with fluorescent secondary antibodies (VectaFluor Duet Immunofluorescence Double Labeling Kit, cat# DK-8818 Vector Laboratories, Newark, CA, USA) for one hour at room temperature. Finally, slides were mounted with Vectashield Antifade Mounting Medium containing DAPI (cat# H-1800-10 Vector Laboratories) to label cell nuclei. This staining allowed us to visualize neuronal structure and motor protein distribution under a fluorescence microscope.

### 2.2. In Vivo Studies

#### 2.2.1. MPTP Exposure in Mice

Young adult male CD57/BL6 mice (weighing between 25 and 30 g, obtained from Charles River, Wilmington, MA, USA) were housed in the animal facility under standard conditions, which included a 12-h light–dark cycle, a temperature of 23 °C, and 55% relative humidity. The mice had unrestricted access to food and water. Animal handling and care adhered to the guidelines provided by the National Institutes of Health (NIH, Bethesda, MD, USA) in the Guide for the Care and Use of Laboratory Animals (NIH publication 80-23, revised 1996). This study protocol was reviewed and approved by the Institutional Animal Care and Use Committee (IACUC) at the Ralph H. Johnson VA Medical Center in Charleston, SC, USA (ACORP 700).

To study how calpain-2 inhibition affects neurodegeneration in a living system, we used a mouse model of Parkinson-like neurotoxicity induced by MPTP [9,10,38]. Young adult C57BL/6 mice were divided into three groups: (1) Vehicle control, (2) MPTP treatment, and (3) MPTP + calpain-2 inhibitor (zLLY-CH_2_F). MPTP (cat# M0896, SIGMA, St. Louis, MO, USA) was dissolved in sterile normal saline and administered intraperitoneally (i.p.) at a dose of 25 mg/kg body weight once daily for five consecutive days. This dosing schedule is known as a sub-chronic regimen and is commonly used to induce neuronal injury.

The calpain-2 inhibitor stock was prepared according to manufacturer guidelines, dissolved in ethanol, and diluted in saline. Mice in the combination group received zLLY-CH_2_F (750 µg/kg) beginning one day after the first MPTP injection, once daily for five days. During MPTP treatment, animals were housed in disposable cages within a chemical fume hood for safety and then returned to standard housing after 72 h post-MPTP treatment.

#### 2.2.2. Tissue Collection and Processing

Mice were euthanized 7–10 days after MPTP exposure. The brain and spinal cord were carefully removed and fixed in 4% paraformaldehyde [41]. Spinal cords were embedded in paraffin for longitudinal sections. Sections were mounted onto glass slides for staining.

#### 2.2.3. Immunohistochemistry

Slides with paraffin spinal cord sections were deparaffinized by immersion in xylene for 3 h, followed by a graded descending series of alcohols (100–50%) and rinsing in deionized water [9,38]. After washing in PBS, antigen retrieval was performed by heating sections in sodium citrate buffer (10 mM, pH 6) for 1 h in an oven. Sections were permeabilized with PBST (PBS with 0.01% Triton X-100) and blocked with 10% normal serum to reduce nonspecific binding. Slides were then incubated overnight at 4 °C with primary antibodies against dynein and kinesin. The next day, sections were washed and incubated with DyLight 594 secondary antibody (1:100, cat# DI-2594, Vector Laboratories) for 1 h at room temperature. Slides were mounted with vectashield vibrance antifade medium containing DAPI (cat# H-1800, Vector Laboratories).

#### 2.2.4. Image Acquisition and Analysis

Images were captured using an Olympus IX73 microscope (Olympus Corporation, Tokyo, Japan). ImageJ/Fiji (NIH Image 1.54r) software was used to measure: (a) neurite length using the NeuronJ plugin, and (b) integrated fiber density using 8-bit images and selecting a consistent region of interest (ROI). Quantitative measurements were performed using identical image settings for all groups.

#### 2.2.5. Statistical Analysis

All statistical analyses were performed using GraphPad Prism (v6.0) and Microsoft Excel [38]. Data are presented as mean ± standard deviation (SD). Depending on the experiment, statistical comparisons were made using either a two-tailed Student’s *t*-test or one-way ANOVA followed by Bonferroni post hoc testing. A *p*-value < 0.05 was considered statistically significant.

## 3. Results

### 3.1. Determination of Optimal Treatment Conditions in VSC 4.1 Motoneuron Cells

**Figure 1:**
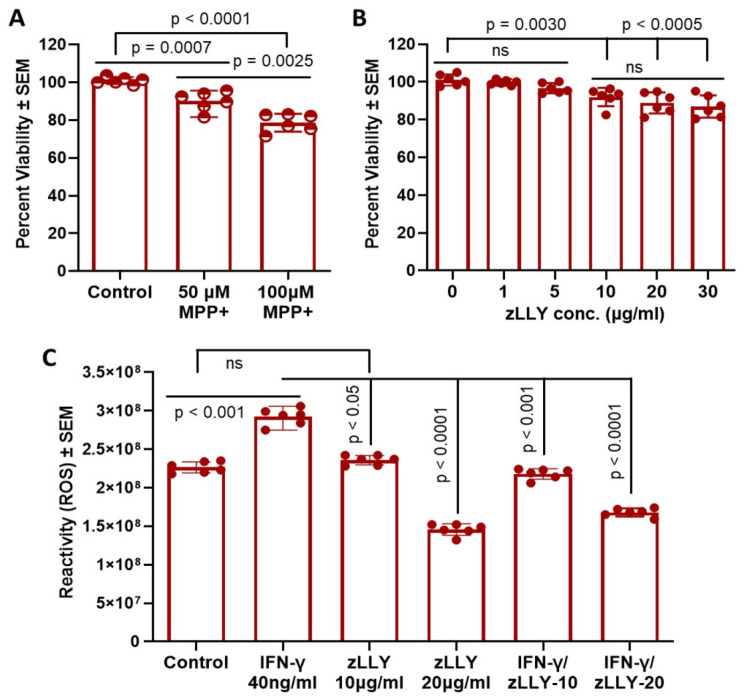
Experimental Treatment Conditions for VSC 4.1 Motoneuron Cells. (**A**) VSC 4.1 motoneuron cells were treated with a range of MPP^+^ concentrations for the MTS viability assay. (**B**) Cells were similarly exposed to varying doses of the calpain-2 inhibitor zLLYCH_2_F (zLLY) for MTS analysis. (**C**) For the ROS assay, cells were treated with IFN-γ in the presence or absence of the calpain-2 inhibitor. For subsequent in vitro experiments, 100 µM MPP^+^ and 20 µg/mL zLLYCH_2_F were selected as the working concentration based on these treatment conditions. *p* < 0.05 versus control. N = 3; ns: not significant.

To establish the effective experimental conditions for subsequent in vitro analyses, VSC 4.1 motoneuron cells were treated with a range of MPP^+^ concentrations to assess cytotoxicity and cellular viability using the MTS assay (Figure 1A). Based on this assay, 100 µM concentration of MPP^+^ was selected for in vitro studies. Similarly, cells were exposed to increasing concentrations of the calpain-2 inhibitor zLLYCH_2_F (zLLY) to determine a non-toxic, effective dose (Figure 1B). For in vitro studies, zLLY at 10 and 20 µg/mL was selected.

Reactive oxygen species (ROS) production was also evaluated in the presence of IFN-γ with or without zLLYCH_2_F (Figure 1C). Based on these data, 20 µg/mL zLLYCH_2_F was selected for subsequent experiments. These doses were sufficient to induce measurable stress responses in VSC 4.1 cells without causing excessive cytotoxicity, establishing a reliable model to study the effects of MPP^+^ neurotoxicity and the protective role of calpain-2 inhibition.

### 3.2. Calpain-2 Inhibition Enhances Neurite Outgrowth in MPP^+^-Treated Cells

**Figure 2:**
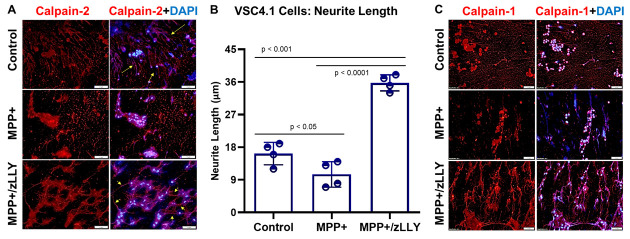
Immunofluorescence Analysis of Calpain-2 Expression and Neurite Morphology in VSC 4.1 Motoneuron Cells. (**A**) VSC 4.1 cells were treated for 24 h with 100 µM MPP^+^ or with the combination of MPP^+^ and 20 µg/mL zLLYCH_2_F (calpain-2 inhibitor, zLLY). Following ice-cold methanol fixation, cells were immunostained for calpain-2, and nuclei were counterstained with DAPI. Arrowheads indicate neurite extensions observed under combination treatment. (**B**) Quantification of neurite length using ImageJ shows increased neurite measurements in the MPP^+^ + zLLYCH_2_F group compared with the control and MPP^+^-only groups. Data are presented as mean ± SEM. N = 3. (**C**) Cells were also immunostained for calpain-1, and nuclei were counterstained with DAPI. Bar = 50 μM

Immunofluorescence staining revealed that MPP^+^ treatment significantly increased calpain-2 expression and led to observable alterations in neurite morphology, with fewer and shorter neuronal extensions compared to control cells (Figure 2A). Remarkably, co-treatment with zLLYCH_2_F markedly improved neurite outgrowth, as highlighted by the prominent neurite extensions (arrowheads) in the MPP^+^ + zLLYCH_2_F group. Quantitative analysis using ImageJ confirmed that neurite length was significantly increased in the combination treatment group relative to both MPP^+^-only and control groups (Figure 2B), suggesting that calpain-2 inhibition not only protects motoneurons from MPP^+^-induced stress but also promotes structural recovery and potentially enhances neuronal connectivity.

### 3.3. MPP^+^-Induced Redistribution of Kinesin and Dynein in Motoneuron Cells

**Figure 3:**
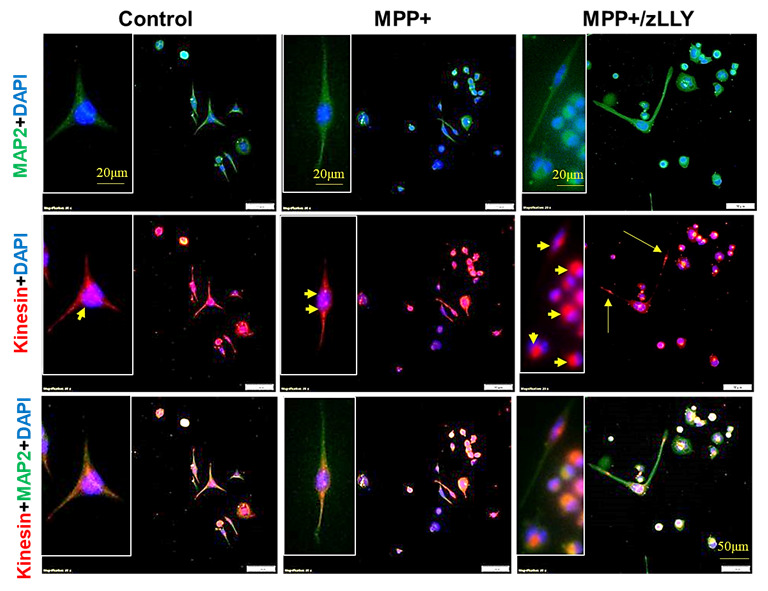
Immunofluorescence Detection of Kinesin and MAP2 in VSC 4.1 Motoneuron Cells. VSC 4.1 cells were immunostained for kinesin and MAP2 to visualize their distribution under different treatment conditions. Control cells (saline-treated) showed labeling in the cytoplasm and cellular processes, with additional nuclear staining for kinesin (arrowhead). Cells treated with MPP^+^ exhibited kinesin immunoreactivity concentrated in the cell body and at the nuclear periphery (arrowheads). In cells treated with the combination of MPP^+^ and the calpain-2 inhibitor zLLYCH_2_F (zLLY), kinesin staining was observed within neuronal fibers (arrows) as well as in the nucleus (arrowheads).

**Figure 4:**
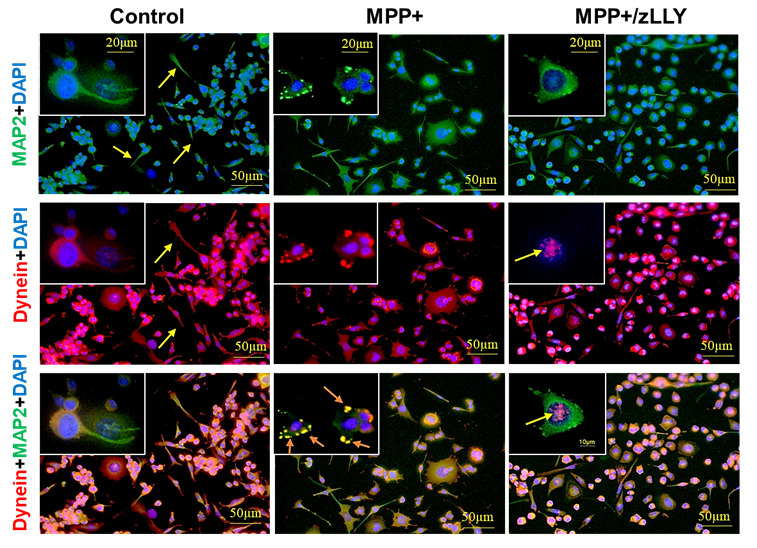
Immunofluorescence Detection of Dynein and MAP2 in VSC 4.1 Motoneuron Cells. VSC 4.1 cells were immunostained for dynein and MAP2 to visualize their localization under different treatment conditions. In control cells, staining for both proteins was observed in the cytoplasm and along neuronal processes. Following MPP^+^ treatment, dynein labeling was detected in the cytoplasm, cell processes, and within the nucleus. Also, some vesicular structures were detected at the periphery of the cells (arrows in inset images). In cells treated with the combination of MPP^+^ and the calpain-2 inhibitor zLLYCH_2_F (zLLY), a distinct dynein immunoreactivity was also evident in the nucleus (arrow).

To further investigate the impact of MPP^+^ on intracellular transport, we examined the localization of kinesin and dynein, key motor proteins involved in anterograde and retrograde vesicular trafficking, respectively (Figure 3 and Figure 4). In control VSC 4.1 cells, kinesin and dynein were distributed throughout the cytoplasm and along neuronal processes, with kinesin also localizing to the nucleus. MPP^+^ exposure caused pronounced alterations: kinesin immunoreactivity accumulated in the cell body and at the nuclear periphery, while dynein labeling extended to the nucleus in addition to the cytoplasm and processes. The dynein immunostaining reveals an accumulation of vesicle-like structures at the cell periphery, especially after exposure to MPP+ (as shown in the magnified view in the inset of Figure 4). It has been demonstrated that dynein mutations lead to the accumulation of intracellular aggregates due to impaired autophagic clearance [42,43]. These observations suggest that MPP^+^ disrupts normal motor protein distribution, potentially impairing vesicular trafficking, and intracellular transport. Notably, co-treatment with zLLYCH_2_F restored kinesin localization within neuronal fibers and enhanced its expression in the nucleus. This calpain-2 inhibitor treatment shows dynein expression in the nucleus and distribution along cellular processes, suggesting that calpain-2 inhibition is an effective strategy to preserve motor protein function and intracellular transport in motoneurons under neurotoxic stress. Interestingly, the vesicular-like aggregates detected at the cell and nuclear peripheries following MPP exposure were absent after calpain-2 inhibition.

### 3.4. Kinesin Fiber Distribution in Spinal Cord Tissue

**Figure 5:**
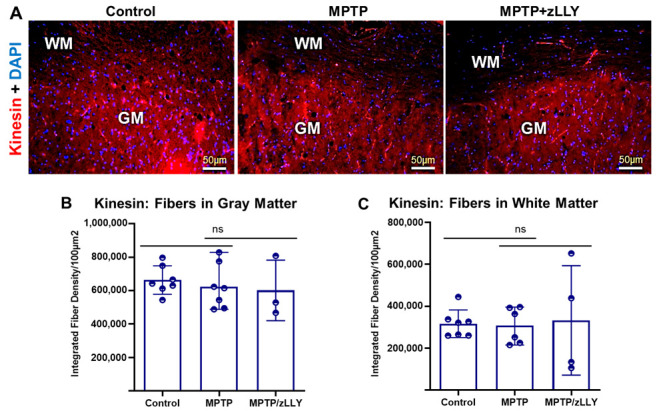
Kinesin Immunostaining and Quantification of Fiber Density in Spinal Cord Tissue. (**A**) Spinal cord sections from each treatment group were immunostained for kinesin to visualize fiber distribution in the gray matter (GM) and white matter (WM). Dense kinesin-positive fibers were observed predominantly in the GM relative to the WM. (**B**,**C**) Quantification of integrated fiber density using ImageJ showed no significant differences in kinesin-stained fiber density among treatment groups in either GM (**B**) or WM (**C**). Data are presented as mean ± SEM. Statistical significance: *p* < 0.05 versus control; ns: not significant.

In vivo analysis of spinal cord sections revealed dense kinesin-positive fibers predominantly in gray matter (GM) relative to white matter (WM) across all treatment groups (Figure 5A). Integrated fiber density measurements demonstrated no significant differences among control, MPTP, and MPTP+zLLYCH_2_F groups in either GM (Figure 5B) or WM (Figure 5C), indicating that MPTP treatment does not substantially disrupt kinesin fiber density at the tissue level. These findings suggest that kinesin distribution is relatively preserved in vivo, contrasting with the pronounced changes observed in dynein and highlighting a potential selective vulnerability of retrograde transport mechanisms to MPTP neurotoxicity.

### 3.5. Dynein Fiber Density Is Reduced by MPTP and Preserved by zLLYCH_2_F Treatment

**Figure 6:**
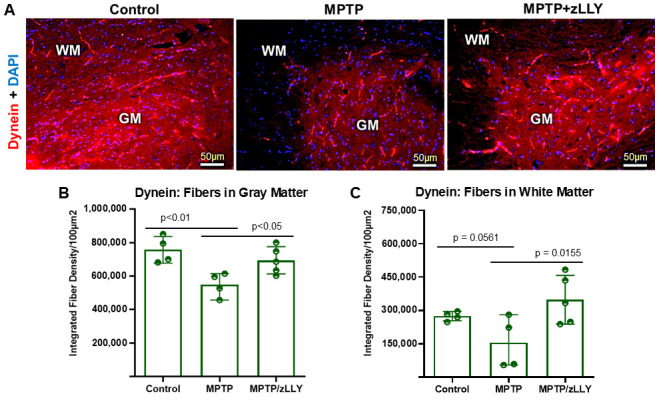
Dynein fiber density in the spinal cord of control, MPTP, and MPTP + zLLYCH_2_F treated mice. (**A**) Representative images of dynein immunostaining show dense fibers in the gray matter (GM) compared to the white matter (WM). MPTP treatment markedly reduces dynein fiber density in both GM and WM, whereas MPTP+ zLLYCH_2_F (zLLY) treatment preserves fiber density similar to control. Quantitative analysis of integrated fiber density using ImageJ confirms a significant reduction in dynein staining in MPTP-treated mice in GM (**B**) and WM (**C**), while MPTP+ zLLYCH_2_F treatment mitigates these effects. Data are presented as mean ± SEM. Statistical significance: *p* < 0.05 versus control; *p* < 0.05 versus MPTP.

Dynein immunostaining in spinal cord sections revealed a marked reduction in fiber density in both GM and WM following MPTP treatment (Figure 6A). Quantitative analysis confirmed a significant decrease in integrated dynein fiber density in MPTP-treated mice compared with control (Figure 6B,C; *p* < 0.05), consistent with impaired retrograde transport. Importantly, co-treatment with zLLYCH_2_F restored dynein fiber density to levels comparable with control, demonstrating that calpain-2 inhibition effectively mitigates MPTP-induced loss of dynein fibers in vivo. These data indicate that dynein-mediated retrograde transport is selectively susceptible to MPTP-induced neurodegeneration, and that calpain-2 represents a key mediator of this effect.

### 3.6. Schematic Overview of Motor Protein Dysfunction and Rescue

**Figure 7:**
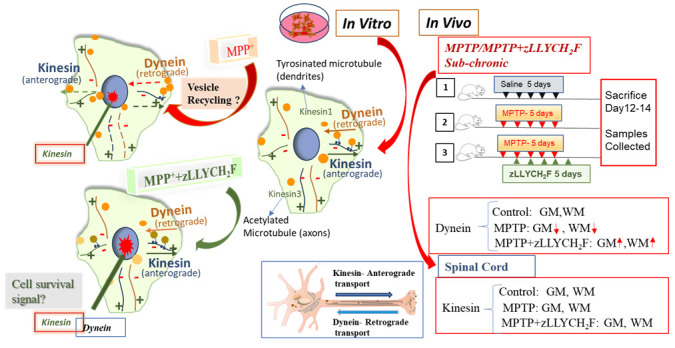
Schematic illustrating the effects of MPP+ and MPTP on motor protein function and vesicular transport, and the protective role of calpain-2 inhibition. In VSC4.1 cells, MPP+ treatment induces accumulation of vesicles at the cell periphery, indicating disrupted vesicular recycling likely due to dynein dysfunction. Vesicle-like structures also appear near the nuclear periphery, suggesting kinesin impairment. Co-treatment with the calpain-2 inhibitor zLLYCH_2_F (zLLY) restores dynein and kinesin function, normalizing vesicle distribution. In vivo, spinal cord sections from MPTP-treated mice show reduced dynein fiber density in both gray matter (GM) and white matter (WM) compared to control (saline) mice, while MPTP+ zLLYCH_2_F treatment preserves fiber density. Integrated fiber density measurements using ImageJ confirm these observations, highlighting the protective effect of calpain-2 inhibition on motor protein function. Small red arrows; upward (increased), downward (decreased).

A schematic model summarizes the in vitro and in vivo findings (Figure 7). In VSC 4.1 cells, MPP^+^ induces vesicular accumulation at the cell periphery, reflecting dynein dysfunction, while perinuclear vesicle accumulation suggests impairment of kinesin-mediated transport. Calpain-2 inhibition with zLLYCH_2_F normalizes vesicle distribution and restores proper motor protein localization, supporting both anterograde and retrograde transport. In vivo, MPTP exposure reduces dynein fiber density in spinal cord GM and WM, whereas co-treatment with zLLYCH_2_F preserves dynein integrity, consistent with the in vitro observations. Overall, these results highlight the critical role of calpain-2 in mediating motor protein dysfunction and suggest that its inhibition represents a promising strategy to protect neuronal transport mechanisms under neurotoxic conditions.

## 4. Discussion

The present study demonstrates that calpain-2 is a critical mediator of motor protein dysfunction and intracellular transport impairment in motoneurons exposed to MPP^+^/MPTP neurotoxicity. Using complementary in vitro and in vivo models, we show that MPP^+^ treatment disrupts the localization of kinesin and dynein, diminishes neurite integrity, and perturbs vesicular distribution, effects that are substantially reversed by selective calpain-2 inhibition. These findings provide new mechanistic insight into how neurotoxin-induced stress compromises transport systems in motoneurons and identifies calpain-2 as a promising therapeutic target for maintaining neuronal architecture and axonal transport under degenerative conditions.

Calpain activation has long been associated with cytoskeletal breakdown, microtubule destabilization, and axonal degeneration [29,44,45], yet its specific contribution to motor protein dysregulation has remained inadequately defined. Our observations that MPP^+^ increases calpain-2 expression and coincides with neurite retraction strongly support a model in which calpain-2 contributes directly to cytoskeletal impairment and reduced axonal transport capacity (Figure 2). On the other hand, little to no change in calpain-1 expression was observed. The restoration of neurite outgrowth by zLLYCH_2_F suggests that calpain-2 inhibition promotes structural recovery, potentially by stabilizing microtubules and limiting proteolytic degradation of cytoskeletal components. This is consistent with studies showing that calpain-2 inhibition enhances neurite regeneration and axonal stability in other neurotoxic or injury models [30,46].

A key finding of this study is that MPP^+^ caused distinct and predictable shifts in motor protein localization: kinesin aggregated around the nucleus, while dynein accumulated abnormally in both the nucleus and soma. These changes suggest that MPP^+^ disrupts microtubule tracks and impairs motor protein binding and mobility, echoing observations in dopaminergic neurons exposed to mitochondrial toxins [25,47,48]. Because kinesin and dynein cargo trafficking is essential for mitochondrial distribution, autophagosome clearance, and synaptic function, the observed redistribution likely reflects early-stage transport collapse before overt cell death.

Importantly, zLLYCH_2_F not only promotes neurite outgrowth but also normalized motor protein distribution, restoring kinesin to neurites and maintaining dynein localization along processes. This indicates that the proteolytic activity of calpain-2 may directly or indirectly influence the organization of microtubules and the recruitment of motor proteins to axonal pathways. Additionally, the localization of both motor proteins in the cell nucleus, following calpain-2 inhibition, suggests a possible induction of survival signals. Whether calpain-2 acts by proteolyzing cytoskeletal scaffolding proteins, by altering Ca^2+^-dependent signaling pathways, or by modifying microtubule dynamics remains an important question for future investigation.

Our in vivo analyses revealed a striking contrast between kinesin and dynein vulnerability to MPTP exposure. While kinesin fiber density in gray and white matter remained relatively preserved across treatment groups, dynein fibers were significantly reduced in both gray and white matter by MPTP and rescued by zLLYCH_2_F. This selective susceptibility of dynein is noteworthy. Retrograde transport is tightly linked to cellular stress responses, neurotrophic signaling, and clearance of damaged organelles [49,50,51]. Thus, dynein dysfunction could amplify neurodegeneration by impairing critical feedback signaling from distal axons

The preservation of dynein fibers with calpain-2 inhibition suggests that calpain-2 may disproportionately affect retrograde transport machinery. This selective sensitivity aligns with emerging evidence that dynein complexes are more vulnerable to oxidative damage, proteolytic cleavage, and microtubule destabilization than kinesin [52,53]. Our findings strengthen the view that retrograde transport failure is an early pathophysiological hallmark of MPTP-induced spinal cord pathology and that calpain-2 is a key molecular regulator of this process.

The alignment between VSC 4.1 cell findings and spinal cord tissue data strengthens the physiological relevance of calpain-mediated transport dysfunction. The in vitro model permitted detailed mechanistic evaluation of motor protein redistribution and neurite architecture, revealing rapid, stress-induced alterations that precede cell death. In vivo, the preservation of dynein fiber density by zLLYCH_2_F confirms that calpain-2 activity contributes to neurotoxin-driven transport deficits at the tissue level. The convergence of these results supports a model in which calpain-2 acts as a central node linking oxidative stress, cytoskeletal destabilization, and transport collapse.

Axonal transport failure is increasingly recognized as a reversible and early event in neurodegenerative conditions [54,55], making it an attractive therapeutic target. Our data support the concept that preserving transport machinery may forestall downstream degenerative processes. Calpain-2 inhibition appears to serve not only a protective role but also a restorative one, improving neurite structure and normalizing intracellular organization. Given that many current neuroprotective strategies target late-stage events such as apoptosis [56,57], interventions aimed at transport preservation may offer broader therapeutic windows. Moreover, calpain inhibitors have shown efficacy in models of ALS, spinal cord injury, and PD [10,11,58,59]. The present findings extend those observations by demonstrating a specific impact on transport-related proteins in motoneurons, suggesting that calpain-2 inhibitors may have utility beyond traditional dopaminergic models.

The selective vulnerability of dynein fibers to MPTP highlights retrograde transport as a critical early failure point in neurodegeneration. Unlike kinesin, dynein is essential for transporting survival signals (like neurotrophic factors) and damaged organelles from the axon back to the soma [60,61]. Its disruption leaves neurons blind to distal damage and unable to clear toxic waste, accelerating cell death. This specific susceptibility likely stems from dynein’s molecular complexity. The large dynein-dynactin complex, reliant on numerous adapter proteins, is a prime target for calpain-2 cleavage under stress [62,63]. By degrading these regulatory components or their microtubule anchors, calpain-2 may preferentially dismantle retrograde transport. The inhibitor zLLYCH_2_F could protect dynein by blocking this targeted proteolysis. Thus, calpain-2 inhibition offers more than cytoskeletal stabilization; it specifically preserves the neuron’s vital communication and recycling system. Thus, protecting dynein maintains the retrograde stress-response pathway, breaking the cycle where transport failure leads to amplified degeneration.

Our findings that calpain-2 directly disrupts dynein and kinesin localization and function align with a growing consensus that calpain-mediated axonal transport failure is not merely a secondary consequence, but a common convergent mechanism in neurodegeneration. Multiple lines of evidence from diverse disease models indicate that, regardless of the initial genetic or environmental insult, the hyperactivation of calpain proteases represents a critical point of convergence that directly compromises the microtubule-based transport system [64,65,66]. In amyotrophic lateral sclerosis (ALS), calpain overactivation is a well-documented feature, driven by pathogenic proteins such as mutant SOD1, TDP-43, or the dipeptide repeats of C9orf72 [67,68,69]. This activation leads to the cleavage of specific cytoskeletal components like neurofilament subunits and spectrin, as well as motor protein adaptors and regulators. These proteolytic events destabilize the microtubule network and uncouple motor proteins from their cargoes, directly impairing both anterograde and retrograde trafficking. The particular vulnerability of retrograde, dynein-dependent transport that we observed in our model is especially significant, as it mirrors the “dying-back” axonopathy central to ALS pathophysiology. The failure of retrograde survival signaling and the impaired clearance of damaged organelles, both dynein-dependent processes, are thought to be critical early steps in motor neuron degeneration.

Similarly, in peripheral neuropathies, such as those induced by diabetes or chemotherapeutic agents like paclitaxel, metabolic and toxic stress converge on calpain activation [70,71]. Hyperglycemia or drug toxicity induces oxidative stress and calcium dyshomeostasis, triggering calpain-mediated proteolysis of microtubule-associated proteins (e.g., tau, MAP2) [72,73]. This destabilization disrupts the efficient trafficking of mitochondria to energy-demanding distal axons and impedes the retrograde transport of neurotrophic factors, ultimately driving a distal-to-proximal axonal degeneration. While the primary triggers of these diseases are diverse, they frequently converge on the dysregulation of intracellular calcium homeostasis, leading to sustained calpain activation. Calpain-2, which is often associated with longer-term pathological processes rather than acute signaling, appears to play a particularly detrimental role. It cleaves a conserved set of substrates integral to axonal integrity: the structural components of the cytoskeletal tracks (e.g., spectrin, neurofilaments) and the motor complexes themselves or their anchoring systems, such as Rab11 [74]. Rab11, a member of the small GTPase family, regulates intracellular vesicle trafficking. It modulates the processes of α-synuclein secretion and aggregation, which are critical mechanisms in the progression of α-synuclein pathology in Parkinson’s disease and also in other synucleinopathies [75].

This dual attack on both the highway and the vehicles leads to the common functional outcome: the collapse of directed intracellular transport. Consequently, calpain-2 emerges as a key pathological node, a shared amplifier of damage across etiologically distinct conditions. This centrality makes it a highly promising broad-spectrum therapeutic target. Inhibiting calpain-2 offers a strategic approach to preserve axonal integrity, maintain neuronal connectivity, and potentially slow progression across a spectrum of neurodegenerative diseases characterized by early transport deficits.

This study identifies Calpain-2 inhibition as a compelling therapeutic strategy for preserving axonal transport. Translating this finding requires a balanced assessment of the candidate zLLYCH_2_F. Its primary strength is selectivity for calpain-2 over calpain-1, potentially reducing side effects by sparing physiological calpain functions. Furthermore, its ability to restore neurite architecture and normalize motor protein distribution suggests true disease-modifying potential, targeting an early pathological convergence point. However, as a peptide-based inhibitor, zLLYCH_2_F faces significant pharmacokinetic hurdles, including poor oral bioavailability and uncertain blood–brain barrier penetration, which must be addressed through advanced formulation or delivery methods.

To maximize clinical potential and mitigate risks, a rational combination therapy is strongly advised. Since calpain activation is a downstream consequence of oxidative stress, co-administering zLLYCH_2_F with a mitochondrial-targeted agent (e.g., MitoQ) offers a powerful, synergistic approach. This dual strategy would simultaneously reduce the initiating oxidative insult and protect the cytoskeletal transport machinery, potentially allowing for lower, safer doses of each drug while achieving superior neuroprotection compared to monotherapy. In conclusion, the future clinical utility of zLLYCH_2_F hinges on overcoming its delivery limitations and embracing a multi-target therapeutic paradigm. Future work must prioritize optimizing its drug-like properties and rigorously testing its efficacy in chronic disease models as part of a combination regimen. This integrated strategy, firmly rooted in the mechanistic link between calpain-2 and transport failure, represents the most viable path for developing an effective treatment to halt neurodegenerative progression.

Several limitations warrant discussion. First, while the MPP^+^/MPTP paradigm is well established, it does not fully recapitulate chronic or progressive aspects of human neurodegenerative disease. Future studies could incorporate additional models, including genetic systems or chronic toxin exposure. Second, although our findings implicate calpain-2, crosstalk with calpain-1 or other proteases may contribute to the observed effects. Mechanistic experiments examining substrate cleavage, microtubule stability, and live-cell transport dynamics would strengthen causal conclusions. Future studies may focus on (1) Live-cell imaging of vesicle transport to quantify calpain-dependent changes in trafficking velocity and directionality; (2) Proteomic analysis of cytoskeletal and motor protein complexes to identify calpain-2 substrates; (3) Longitudinal in vivo studies to determine whether sustained calpain inhibition prevents functional deficits or neurodegeneration; and (4) Therapeutic combination strategies, integrating calpain inhibitors with antioxidants or mitochondrial protectants to enhance neuroprotection.

Collectively, this study provides strong evidence that calpain-2 plays a critical role in neurotoxin-induced disruption of intracellular transport in motoneurons. By preventing motor protein mislocalization, preserving dynein fiber integrity, and enhancing neurite structure, calpain-2 inhibition emerges as a promising approach for maintaining axonal transport under neurotoxic stress. These findings establish a mechanistic foundation for future therapeutic strategies aimed at stabilizing neuronal transport systems, a central and potentially disease-modifying target in neurodegeneration.

## 5. Conclusions

In summary, this study establishes a critical role for the protease calpain-2 in mediating motor protein dysfunction and intracellular transport deficits in motoneurons under neurotoxic stress. Using both in vitro and in vivo models of MPP^+^/MPTP-induced neurotoxicity, we demonstrate that calpain-2 activation contributes to neurite retraction, the aberrant redistribution of the key transport motors kinesin and dynein, and the accumulation of vesicular structures. Strikingly, selective pharmacological inhibition of calpain-2 with zLLYCH_2_F effectively counteracted these pathological changes. It restored neurite outgrowth, normalized the localization of motor proteins within neuronal processes, and, in vivo, specifically prevented the MPTP-induced loss of dynein-positive fibers in spinal cord gray and white matter.

A key and consistent finding is the selective vulnerability of dynein-mediated retrograde transport to MPTP/MPP^+^ toxicity and its rescue by calpain-2 inhibition. This highlights retrograde transport failure as an early pathological event and identifies calpain-2 as a pivotal regulator of this process. The convergence of our cellular and tissue-level observations supports a mechanistic model whereby neurotoxin-induced stress activates calpain-2, leading to cytoskeletal destabilization and the subsequent disruption of motor protein function and intracellular trafficking.

Our findings position calpain-2 inhibition not merely as a cytoprotective strategy but as a means to actively promote structural recovery and preserve essential axonal transport mechanisms. By targeting an early, convergent pathway in neurodegeneration, axonal transport dysfunction, this approach offers a promising therapeutic avenue with a potentially wider treatment window. Future research should focus on elucidating the precise substrates of calpain-2 in this context, validating these effects in chronic or genetic models, and exploring the functional and long-term neuroprotective benefits of sustaining transport integrity through calpain-2 modulation.

## Data Availability

The original contributions presented in this study are included in the article. Further inquiries can be directed to the corresponding author.
